# Comparative cochlear transcriptomics of echolocating bats provides new insights into different nervous activities of CF bat species

**DOI:** 10.1038/s41598-018-34333-7

**Published:** 2018-10-29

**Authors:** Hui Wang, Hanbo Zhao, Xiaobin Huang, Keping Sun, Jiang Feng

**Affiliations:** 1 Jilin Provincial Key Laboratory of Animal Resource Conservation and Utilization, Northeast Normal University, Changchun, 130117 China; 2 Jilin Agricultural University, Changchun, 130118 China

## Abstract

The molecular mechanisms used by echolocating bats to deal with different ultrasonic signals remain to be revealed. Here, we utilised RNA-Seq data to conduct comparative cochlear transcriptomics to assess the variation of gene expression among bats with three types of echolocation: constant-frequency (CF) bats, frequency-modulated (FM) bats and click bats. Our results suggest larger differences in gene expression between CF and click bats than between CF and FM bats and small differences between FM and click bats. We identified 426 and 1,504 differentially expressed genes (DEGs) by the different methods as functionally important for CF bats, in that they showed consistent upregulation in the cochlea of two CF bats, relative to the levels in click and FM bats. Subsequently, downstream GO and KEGG enrichment analyses indicated that both the 426 and 1,504 gene sets were associated with changes in nervous activities in the cochleae of CF bats. In addition, another set of 1,764 DEGs were identified to have crucial hearing related physiological functions for laryngeally echolocating bats. Our study provides a comprehensive overview of the genetic basis of differences among echolocating bats, revealing different nervous system activities during auditory perception in the cochlea particularly in CF bats.

## Introduction

The divergence of gene expression is an important component of molecular adaptation and an essential way to generate biological diversity^1–3^. Comparisons of gene expression levels within and between species have become a powerful method for studying the genetic basis of phenotypic variation^4^, as well as for studying the evolution of gene regulation^5–7^. Understanding the molecular basis underlying the phenotypic diversity in closely related species is a central goal for evolutionary biology, which can provide new insights into biodiversity^8^.

Echolocating bats are an ideal group in which to examine the differentially expressed genes (DEGs) responsible for the diversity of echolocating types^9,10^. Echolocation is a remarkable and complex phenotypic trait that is well evolved in bats^11^; it is used for navigating in the night sky, such as for avoiding obstacles, orientation and hunting^12,13^. Generally, echolocating bats can be divided into three broad categories according to the dominant frequency ranges and structures of their echolocation calls: constant-frequency (CF) bats, frequency-modulated (FM) bats and click bats^13,14^. Unlike click bats from the genus *Rousettus*, which can produce very brief ultrasonic clicks via the tongue^15,16^, both CF and FM bats emit ultrasonic vocalisations through their larynxes^17,18^.

Among echolocating bats, CF bats have some particularly diverse features. First, the CF component is unique to CF bats, which they possess along with the FM component that is also used by the other two echolocating bat groups^13,19^. Second, CF bats have a special structure called the auditory fovea, a highly expanded frequency representation centralised to the constant frequency, which is present on the basilar membrane^20,21^. This enables CF bats to compensate for Doppler shifts of complete echo signals in order to maintain the audition of echoes within the auditory fovea, which is an important cochlear adaptation^22^. Third, within the foveal area of CF bats, the neurons are highly overrepresented with extraordinarily sharp frequency tuning^22,23^. In contrast, no distinct overrepresentation of neurons with specific optimal frequencies is found in FM bats using pure FM signals^22,24^. We hypothesise that many of these differences may be reflected in cochlear gene expression.

In the last decade, RNA-Seq has emerged as a revolutionary technology for transcriptome analysis^25,26^. In association with this, great interest has developed in using transcriptome tools to identify differences in gene expression between closely related species, such as comparisons between human and nonhuman primates^27^ and between temperate desert and tropical mesic heteromyid rodents^6^. In the case of bats, to the best of our knowledge only one study has determined the genes differentially expressed in the inner ear between echolocating bats (FM type) and nonecholocating bats using RNA-Seq^28^. Researchers found that, for genes upregulated in Rickett’s big-footed bats compared with the levels in greater short-nosed fruit bats, there was significant overrepresentation in biological process categories such as cochlear morphogenesis, inner ear morphogenesis and sensory perception of sounds. However, our knowledge about the genetic bases of mechanisms in bat cochleae that underlie different echolocating types remains limited.

**Figure 1:**
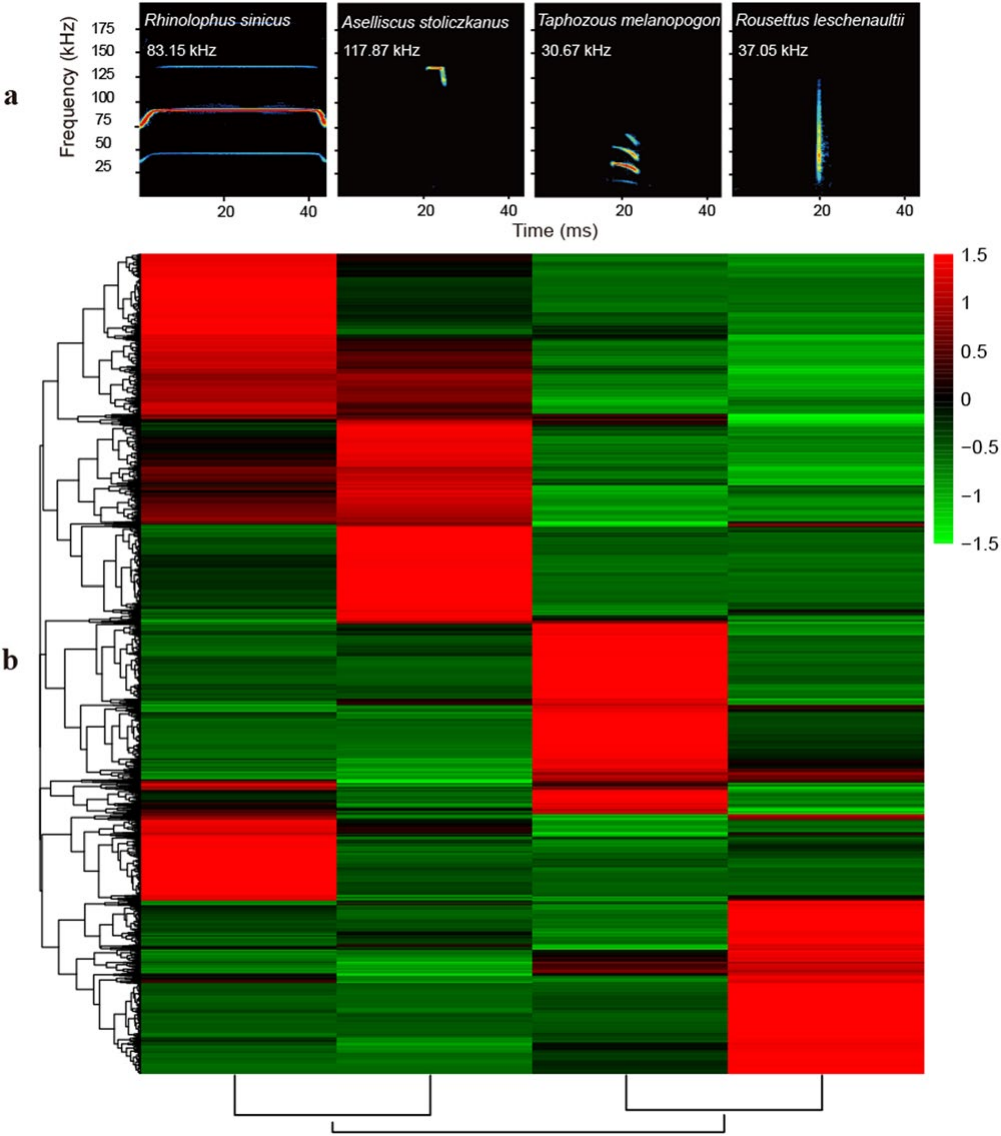
Call types and heatmaps of DEGs for four bat species. (**a**) Spectrogram representation (frequency × time) of echolocation calls of four bat species. Red indicates maximal intensity and the position of the dominant harmonic. Dominant frequency is shown under the name of each species. (**b**) Heatmaps based on differentially expressed genes from six pairwise comparisons of four bat species. Different colours indicate relative expression levels.

To gain insights into the genetic bases underlying the diversity of echolocation evolved by different bat species, in this study, we explored the utility of next-generation sequencing technologies for comparative cochlear transcriptomics to assess the variation in gene expression among three types of echolocating bat: CF, FM and click bats. Taking into consideration the high specialisation of CF bats, two CF bat species, *Rhinolophus sinicus* (Rhinolophidae, dominant frequency: ca. 83.15 kHz) and *Aselliscus stoliczkanus* (Hipposideridae, ca. 117.87 kHz), were included in this study (Fig. 1a). The bats from the above families are sister groups, which are important and representative CF bat families with nasal emitters^13^. For click bats, only one species, *Rousettus leschenaultii* (*Rousettus*, Pteropodidae, ca. 37.05 kHz), was selected because the genus *Rousettus* is the only bat group producing click calls^17,29^. FM bats constitute the majority of echolocating bats and probably evolved several times independently^13^, suggesting the difficulty in selecting representative species. In this study, an FM bat species that is relatively large and sympatric with *R. sinicus*, *A. stoliczkanus* and *R. leschenaultii* was selected, namely, *Taphozous melanopogon* (Emballonuridae, ca. 30.67 kHz). The main purposes of this work are: 1) to detect to what extent the four bat species with different hearing traits differ in gene expression; 2) to determine which hearing-related biological processes or physiological pathways are influenced by the DEGs, especially for those processes that are influenced by genes overexpressed in CF bats; and 3) to elucidate the gene expression profiles based on the bats’ dominant frequencies. This study is expected to provide new insights into the genetic bases underlying the diversity of echolocation evolved by different bat species.

## Results

### Sequence analysis, assembly and functional annotation

After filtering the adaptor sequences, regions containing “N” sequences and low-quality sequences, approximately 34.88, 33.65, 32.61 and 36.45 million clean reads were obtained for *R. sinicus*, *A. stoliczkanus*, *T. melanopogon* and *R. leschenaultii*, respectively (Table 1). The proportions of clean reads among raw tags in each library ranged from 97.48% to 97.92%, suggesting the high quality of the RNA-Seq data available for further analyses.

**Table 1:** Sequencing, assembly and mapping statistics of cochlear samples for four bat species.

	*R. sinicus*	*A. stoliczkanus*	*T. melanopogon*	*R. leschenaultii*
**Sequencing**				
Total Sequences (bp)	5,364,461,900	5,178,733,800	4,996,389,100	5,584,303,600
Total reads (Raw reads)	35,763,079	34,524,892	33,309,261	37,228,691
Clean reads	34,877,043	33,654,723	32,612,027	36,452,156
Ratio of clean/raw	97.53	97.48	97.91	97.92
**Assembly**				
Unigenes	130,381			
N50	1,460			
Max. length	11,491			
Ave. length	837			
**Mapping**				
Total mapped reads (%)	73.15	71.59	70.66	68.81
Unique mapped reads (%)	64.92	63.64	64.73	61.92
Unigenes	67,264	70,135	61,959	65,815

For the reference transcriptome database, a total of 130,381 unigenes were finally yielded, ranging from 201 to 11,491 bp in length, with an N50 of 1,460 bp (Table 1). For the following DEGs analyses of each pairwise comparison, the raw reads of the four species were separately mapped to the 130,381 assembled unigenes, which function as a transcriptome reference database. The results showed that 23,451,494, 20,722,924, 20,460,225 and 22,990,184 reads were mapped for *R. sinicus*, *A. stoliczkanus*, *T. melanopogon* and *R. leschenaultii*, respectively, and the numbers of unique mapped reads were 20,812,887, 18,426,423, 18,466,979 and 20,690,043, with unique mapping rates of 64.92%, 63.64%, 64.73% and 61.92%, respectively. Then, 67,264, 70,135, 61,959 and 65,815 unigenes were identified for *R. sinicus*, *A. stoliczkanus*, *T. melanopogon* and *R. leschenaultii*, respectively. The analyses of the interval distribution of gene expression abundance indicated that the most abundant genes were those with an RPKM value of 1–5 (see Supplementary Fig. S1).

Among the 130,381 unigenes, 67,664 were annotated by at least one annotation database. Specifically, 67,233, 55,192, 42,859, 40,377 and 39,041 unigenes were annotated by the Nr, Swiss-Prot, KOG, KEGG and GO databases (see Supplementary Fig. S2). From the results of KOG annotation in particular, the term of signal transduction mechanisms was the most highly represented (see Supplementary Fig. S3).

### Differential expression analysis

The correlation coefficient between each pair of biological replicates for every species was greater than 0.9 (R^2^ > 0.9) based on the RPKM values, except for two relatively low values for *Role*, 0.77 and 0.78. The results of principal component analysis (PCA) showed four separate groups corresponding to the four bat species (see Supplementary Fig. S4), which were indicative of the sampling having sufficient reproducibility and rationality.

For the four bat species, six pairwise comparisons were conducted for the differential gene expression analyses. For each comparison, namely, *Rhsi* vs. *Role*, *Asst* vs. *Role*, *Rhsi* vs. *Tame*, *Asst* vs. *Tame*, *Asst* vs. *Rhsi* and *Role* vs. *Tame*, the former species always had a higher frequency than the latter (*Rhsi*, *Asst*, *Tame* and *Role* are short for *R. sinicus*, *A. stoliczkanus*, *T. melanopogon* and *R. leschenaultii*, respectively). In terms of the definitions of up- and downregulation of specific genes in each pairwise comparison, an upregulated gene is one that has a higher expression level in the former bat species than in the latter, and vice versa for a downregulated gene. A heatmap of the hierarchical clustering of all DEGs indicated the large differences in gene expression between different bat species (Fig. 1b).

**Figure 2:**
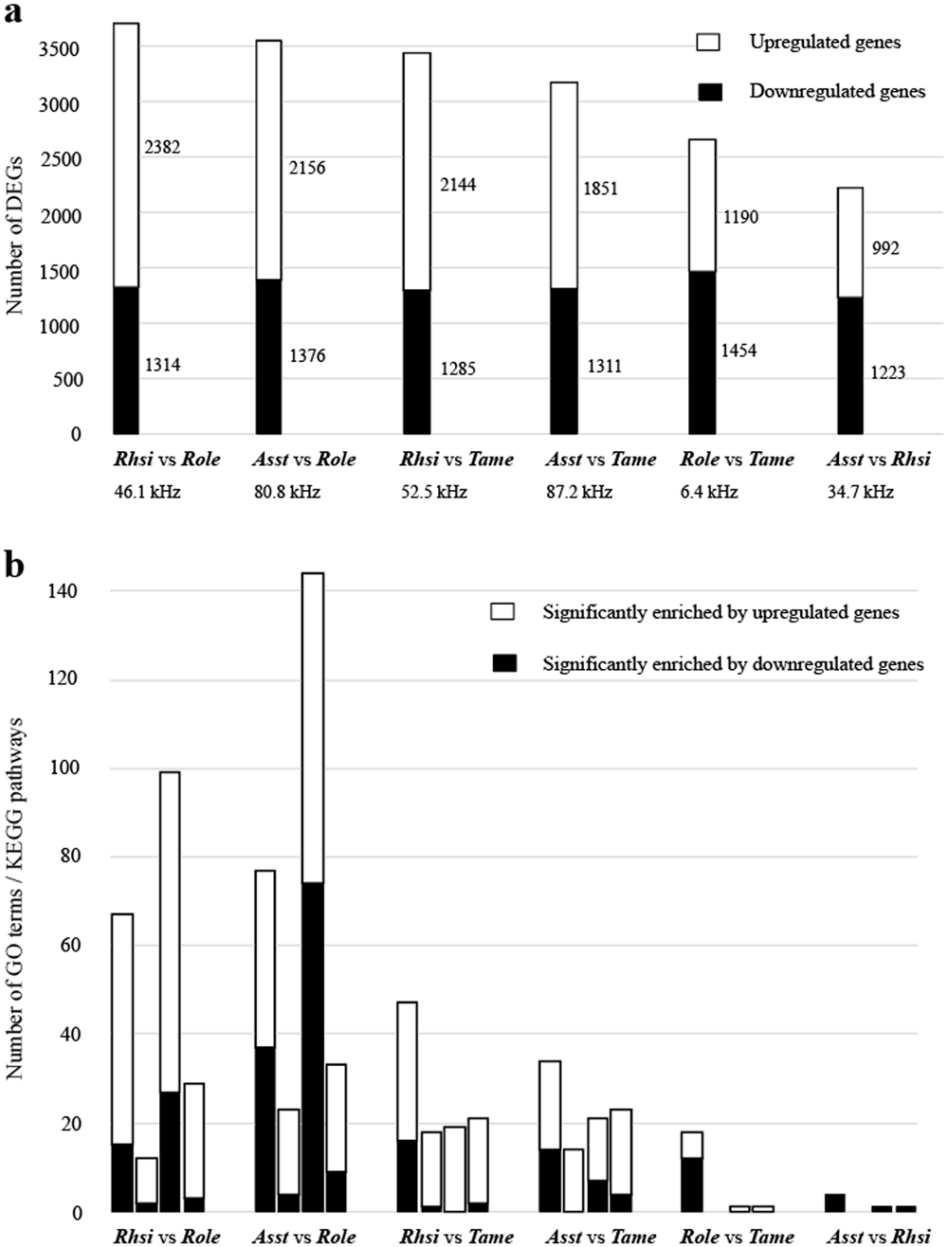
Basic information of DEGs for six pairwise comparisons. (**a**) Numbers of DEGs for six pairwise comparisons. The numbers of up- and downregulated genes are shown beside the bar. The differences in dominant frequency between two bat species are listed under each pairwise comparison. (**b**) Numbers of GO terms and KEGG pathways significantly enriched for up- and downregulated genes. The four bars (from left to right) for each comparison are GO cell component, GO molecular function, GO biology process and KEGG pathways.

An overview of the DEGs indicated that the DEGs in CF vs. Click (*Rhsi* vs. *Role* and *Asst* vs. *Role*) were the most abundant, followed by CF vs. FM (*Rhsi* vs. *Tame* and *Asst* vs. *Tame*) and Click vs. FM (*Role* vs. *Tame*), and the lowest number of DEGs was detected in the CF vs. CF comparison (*Asst* vs. *Rhsi*) (Fig. 2a). In two CF vs. Click and two CF vs. FM comparisons with relatively high frequency differences (>46 kHz), the number of genes upregulated in the cochlea of CF bats with a higher-frequency hearing ability was larger than that of downregulated genes. However, fewer DEGs were detected in the other two comparisons, Click vs. FM and CF vs. CF, with relatively low frequency differences, 6.38 and 34.72 kHz, respectively. Furthermore, the number of upregulated genes was lower than that of downregulated genes in these two comparisons, despite the relatively high-frequency signals emitted by the former bat species.

### GO category and KEGG pathway enrichment analyses

**Figure 3:**
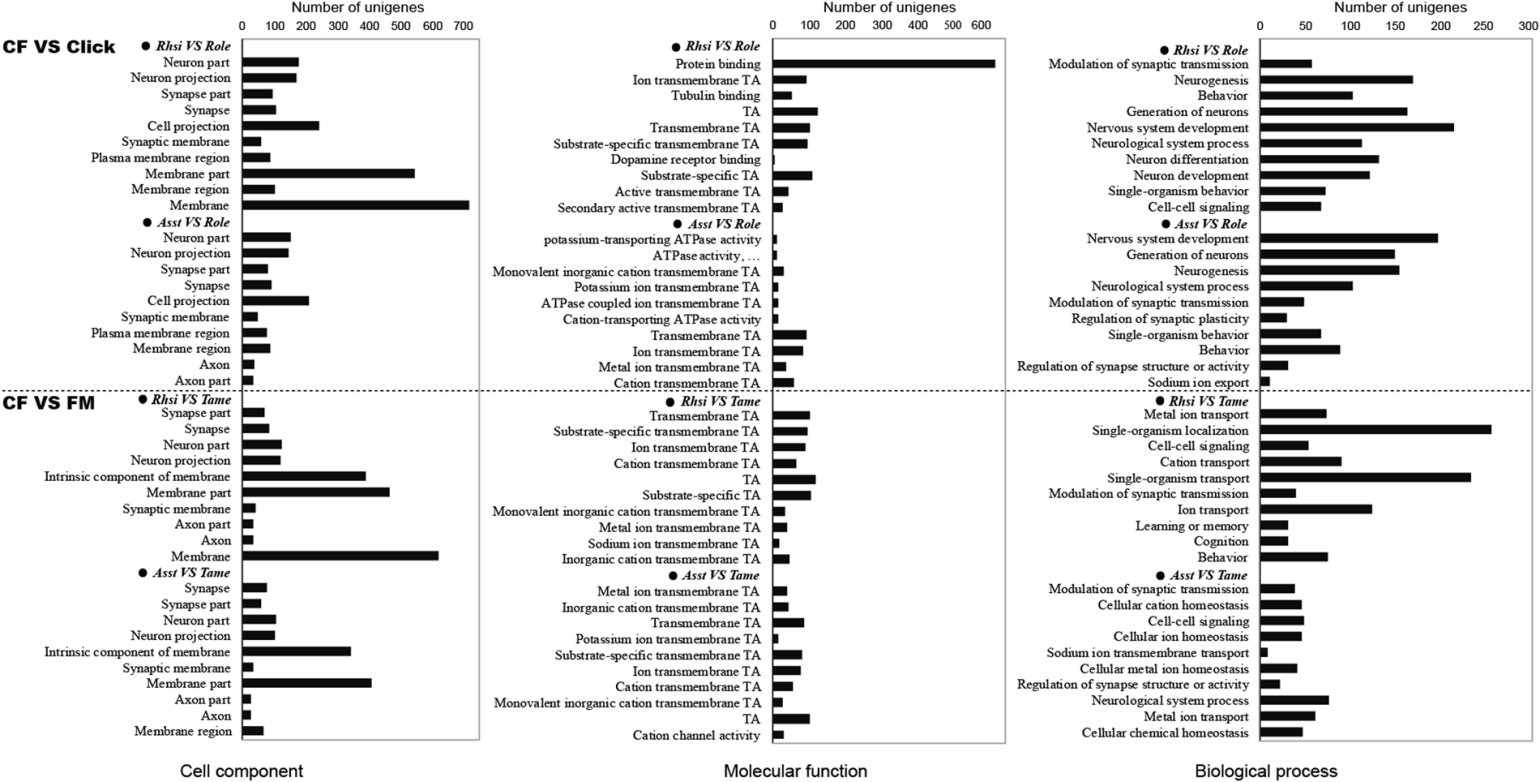
The top 10 GO categories with the most significant *p* values (cell component, molecular function and biological process) were significantly enriched for upregulated genes in two CF vs. Click comparisons (*Rhsi* vs. *Role* and *Asst* vs. *Role*) and two CF vs. FM comparisons (*Rhsi* vs. *Tame* and *Asst* vs. *Tame*).

Numerous GO categories and KEGG pathways were significantly enriched for DEGs from the CF vs. Click and CF vs. FM comparisons; however, fewer terms were significantly enriched for the Click vs. FM and CF vs. CF comparisons (Fig. 2b). In addition, DEGs in the CF vs. Click and CF vs. FM comparisons were significantly enriched in similar terms (see Supplementary Tables S1–S4). In particular, GO categories mostly related to auditory processes were significantly enriched for upregulated genes in CF bat species rather than for downregulated genes in them. The top ten GO terms with the most significant *p* values that were significantly enriched for upregulated genes for the categories of cell component, molecular function and biological process are shown in Fig. 3.

In detail, for the cell component category, upregulated genes in CF bats were significantly enriched in terms that are closely related to various components of the auditory nervous system, such as neuron, synapse, axon and membrane. The three top GO terms with the most significant *p* values were neuron, neuron projection and synapse part for the two CF vs. Click comparisons; and synapse part, synapse and neuron part for the two CF vs. FM comparisons (Fig. 3 and Supplementary Table S1). However, the genes downregulated in CF bats were significantly enriched in terms related to vesicle, cell, cytoplasm and organelle, among others, which are not directly related to the process of auditory perception. All of these findings indicate that there are remarkable differences in nervous activity in the cochlea of CF bats compared with that in the other bats. For Click vs. FM (*Role* vs. *Tame*), 6 and 12 terms were significantly enriched for the up- and downregulated genes, respectively, which were associated with membrane, vesicle and organelle, among others, but not with hearing-related processes.

Regarding the category of molecular function, a variety of transporter activities were represented by the upregulated genes in the CF vs. Click and CF vs. FM comparisons. The three top GO terms with the most significant *p* values were protein binding, ion transmembrane transporter activity and tubulin binding for *Rhsi* vs. *Role*; potassium-transporting ATPase activity, ATPase activity, coupled to transmembrane movement of ions, phosphorylative mechanism and monovalent inorganic cation transmembrane transporter activity for *Asst* vs. *Role*; transmembrane transporter activity, substrate-specific transmembrane transporter activity and ion transmembrane transporter activity for *Rhsi* vs. *Tame*; and metal ion transmembrane transporter activity, inorganic cation transmembrane transporter activity and transmembrane transporter activity for *Asst* vs. *Tame* (Fig. 3 and Supplementary Table S2). Besides, numerous terms related to various ion transporter activities were also represented by the upregulated genes in these four comparisons. Referring to those GO terms enriched for the downregulated genes, these were mostly related to binding function, rather than transporter activity. All of the above results indicate particularly different transporter activity in the cochleae of CF bats. However, no terms in the category of molecular function were represented by those DEGs in the Click vs. FM and CF vs. CF comparisons.

The GO terms for the category of biological process enriched for the upregulated genes also suggested that CF bats exhibit unique auditory processes (Fig. 3 and Supplementary Table S3). Numerous terms related to the nervous system, neurons development, synaptic transmission, cell–cell signaling, homeostasis and ion transport, among others, were all represented by the upregulated genes in the CF vs. Click and CF vs. FM comparisons. The three top GO terms with the most significant *p* values were: modulation of synaptic transmission, neurogenesis and behaviour for *Rhsi* vs. *Role*; nervous system development, generation of neurons and neurogenesis for *Asst* vs. *Role*; metal ion transport, single-organism localisation and cell–cell signaling for *Rhsi* vs. *Tame*; and modulation of synaptic transmission, cellular cation homeostasis and cell–cell signaling for *Asst* vs. *Tame*. Notably, three hearing-related terms, that is, response to auditory stimulus, auditory behaviour and sensory perception, were significantly enriched for the upregulated genes in *Rhsi* vs. *Role*. However, terms enriched for the downregulated genes in those four comparisons were also detected, which were mostly related to other biological processes, such as the regulation of body fluid levels, platelet activation, blood coagulation and immune system processes, which are markedly different from those enriched for the upregulated genes. However, only one term was significantly enriched for the downregulated genes for Click vs. FM and one term for the upregulated genes for CF vs. CF (Supplementary Table S3). These two terms are not related to auditory processes.

**Figure 4:**
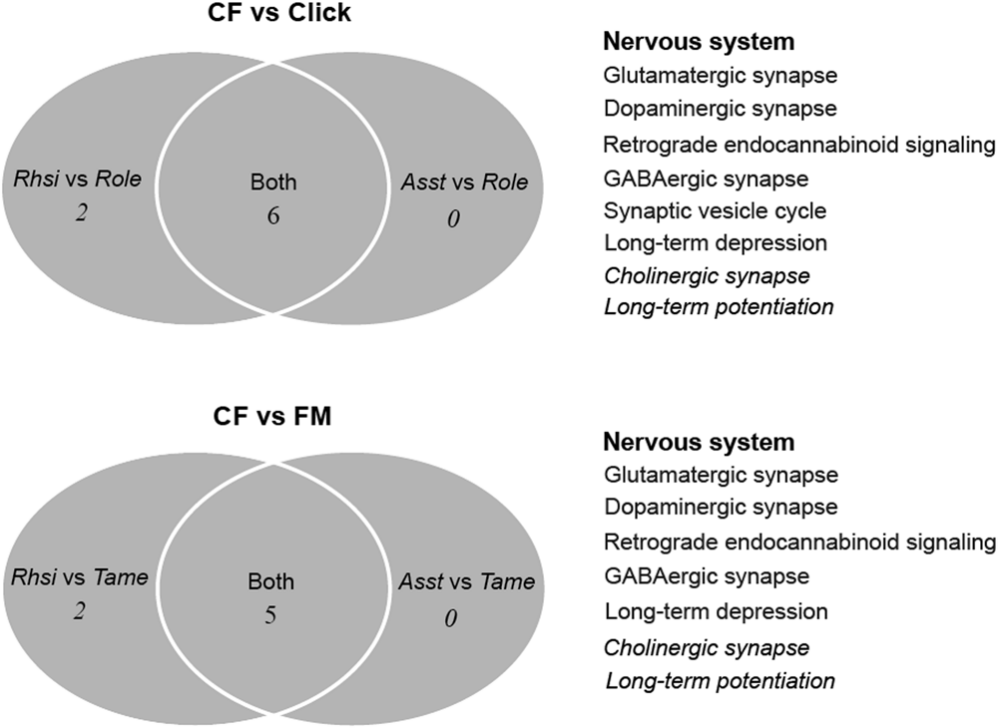
Venn diagram of the nervous system pathways (KEGG) that were significantly enriched for upregulated genes in CF bats relative to those in click and FM bats.

The results of KEGG enrichment analyses of the DEGs are shown in Supplementary Table S4. Numerous pathways were significantly enriched for the upregulated genes in two CF vs. Click and two CF vs. FM comparisons, whereas, fewer pathways were significantly enriched for the downregulated genes. For the upregulated genes, the nervous system was the most represented, followed by signal transduction (only in *Rhsi* vs. *Role* and *Rhsi* vs. *Tame*) (see Supplementary Fig. S5), both of which are closely associated with the process of auditory perception. In particular, for the nervous system, five nervous transduction pathways were all significantly enriched for DEGs in these four comparisons (Fig. 4), namely, glutamatergic synapse (ko04724), dopaminergic synapse (ko04728), GABAergic synapse (ko04727), retrograde endocannabinoid signaling (ko04723) and long-term depression (ko04730), all of which indicate important and unusual synapse transduction in the cochleae of CF bats. For signal transduction, the cAMP signaling pathway (ko04024) was significantly enriched for upregulated genes in both *Rhsi* vs. *Role* and *Rhsi* vs. *Tame*, and the cGMP–PKG signaling pathway (ko04022) was only found in the *Rhsi* vs. *Role* comparison, which suggested that these two signaling pathways are functionally important for *Rhsi*. Moreover, two sensory systems were identified in the two CF vs. Click comparisons, namely, the taste transduction (ko04742) and phototransduction-fly (ko04745) pathways, but these do not have any relationship with hearing phenotypes.

### Clustering analysis of DEGs

**Figure 5:**
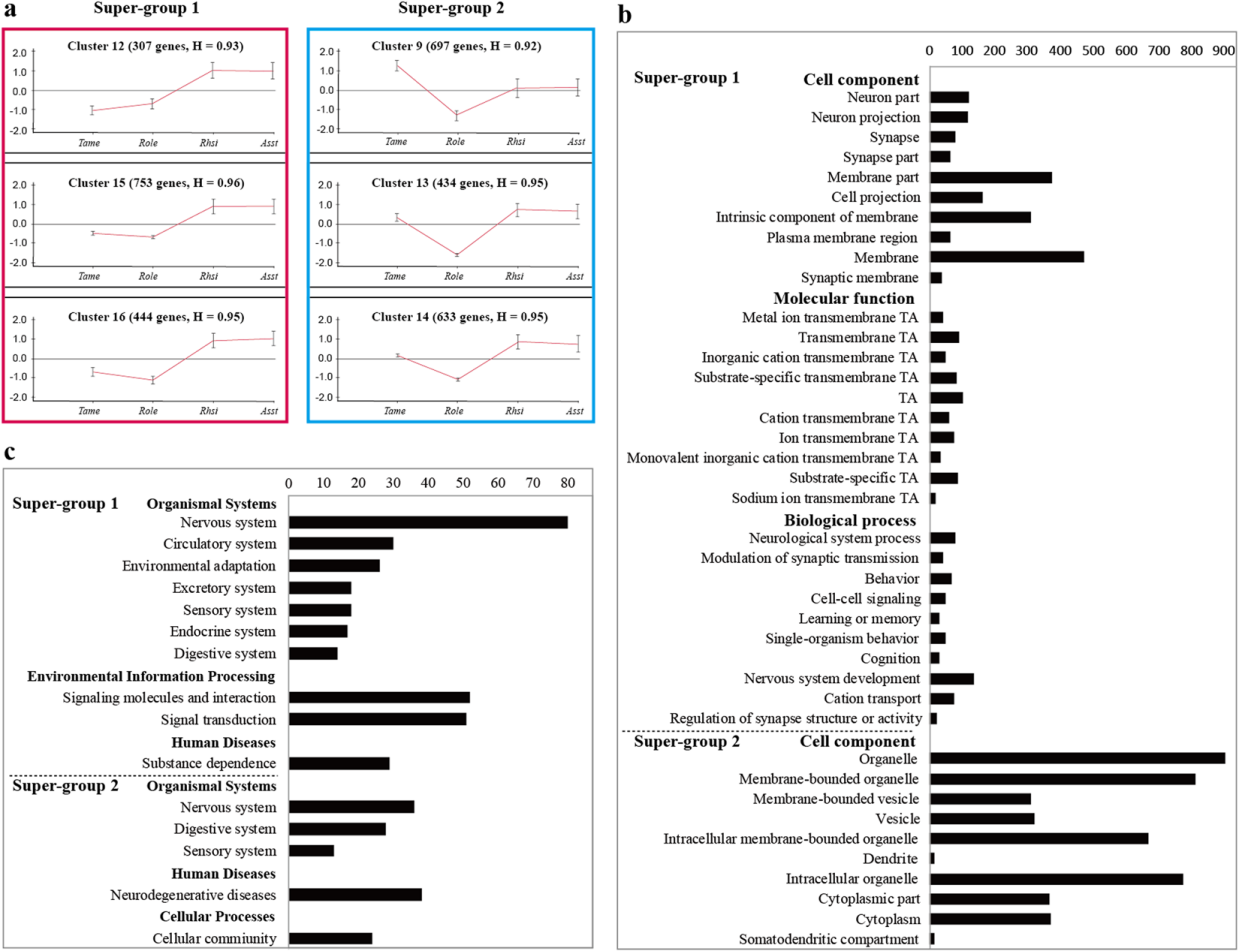
Clustering plots of DEGs and the associated enrichment results. (**a**) Two super-groups of gene clusters based on the expression patterns. In the red super-group 1 (clusters 12, 15 and 16) and blue super-group 2 (clusters 9, 13 and 14), colours represent gene clusters displaying similar expression. The number of genes in each cluster and the homogeneity (H) value are shown at the top of the curve. Each cluster graph displays the mean pattern of expression (red lines) of the genes in it. The x-axis represents species ordered in terms of their dominant frequency from low to high and the y-axis represents log_2_ fold change in gene expression. (**b**) Top 10 GO categories with the most significant *p* values that were significantly enriched for genes from super-group 1 and super-group 2. (**c**) KEGG pathways significantly enriched for DEGs from super-group 1 and super-group 2.

The results of sixteen clusters of 10,630 DEGs according to the expression patterns in the four bat species in the order of their dominant frequencies are shown in Supplementary Fig. S6. Six clusters with homogeneity values greater than 0.9 were found; subsequently, they were divided into two super-groups based on the similarity in expression patterns: super-group 1 (clusters 12, 15 and 16) and super-group 2 (clusters 9, 13 and 14) (Fig. 5a). Downstream functional analyses suggested that both super-group 1 (1,504 DEGs) and super-group 2 (1,764 DEGs) were significantly enriched in several important hearing-related GO categories and KEGG pathways (see Supplementary Table S5).

Moreover, the 1,504 DEGs in super-group 1 exhibited similarly high expression levels in the two CF bats (*Rhsi* and *Asst*), and low expression levels in the FM and click bats (*Tame* and *Role*) (Fig. 5a), suggesting their functional importance in CF bats. Numerous GO categories and KEGG pathways were significantly enriched for the 1,504 DEGs. These results suggest that these 1,504 DEGs play important roles specifically in CF bats, in consideration of their high expression levels in super-group 1.

Upon assessing the above findings in more detail, the GO categories significantly enriched for the 1,504 DEGs (Fig. 5b and Supplementary Table S5) were found to be similar to those categories that were significantly enriched for the genes upregulated in CF bats, as revealed by the six pairwise comparison analyses. For the cell component category, numerous neuron- and synapse-related categories, including neuron part, neuron projection, synapse, synapse part, membrane and synaptic membrane, were significantly enriched. In addition, various transporter activities were also identified for the molecular function category, including metal ion transmembrane transporter activity, cation transmembrane transporter activity and inorganic cation transmembrane transporter activity, along with several types of channel activity, including cation channel activity, ion channel activity and substrate-specific channel activity. Numerous biological process categories that are important for hearing were also significantly enriched, including neurological system process, modulation of synaptic transmission, cell–cell signaling, nervous system development, cation transport and regulation of synapse structure or activity. The results of KEGG enrichment analyses for super-group 1 (Fig. 5c and Supplementary Table S5) were also similar to the enrichment results obtained by the six pairwise comparison analyses. Nervous system was the most overrepresented category, followed by signalling molecules and interaction and signal transduction. Most of these are related to the process of hearing perception.

The 1,764 DEGs in super-group 2 showed relatively high and similar expression levels in FM (*Tame*) and CF bats (*Rhsi* and *Asst*), but low expression levels in click bats (*Role*) (Fig. 5a), which may suggest their importance in FM and CF bats. Furthermore, numerous GO cell component terms were found to be significantly enriched, including organelle, membrane-bounded organelle and dendrite; however, no terms in the categories of molecular function and biological process were significantly enriched (Fig. 5b and Supplementary Table S5). For KEGG analyses, neurodegenerative diseases and nervous system were the two most overrepresented pathways (Fig. 5c). Two pathways involved in the nervous system, retrograde endocannabinoid signaling (ko04723) and dopaminergic synapse (ko04728), were significantly enriched (see Supplementary Table S5).

### Genes important for CF bats

A total of 426 genes were found to be commonly and consistently upregulated in CF bats relative to the levels in FM and click bats, as revealed by the four CF-related pairwise comparisons (see Supplementary Table S6). Interestingly, all of these 426 genes were found in super-group 1 (1,504 DEGs in total), as determined by clustering analysis. In addition, the GO terms and KEGG pathways that were significantly enriched for the 426 genes were similar to those enriched for the 1,504 genes from super-group 1, which indicated that CF bats exhibited particularly different nervous system activities in their cochleae. Both the 426 and 1,504 gene sets identified by the different methods provided strong support that each plays important roles in the cochleae of CF bats.

### Quantitative real-time PCR (qPCR) validation

**Figure 6:**
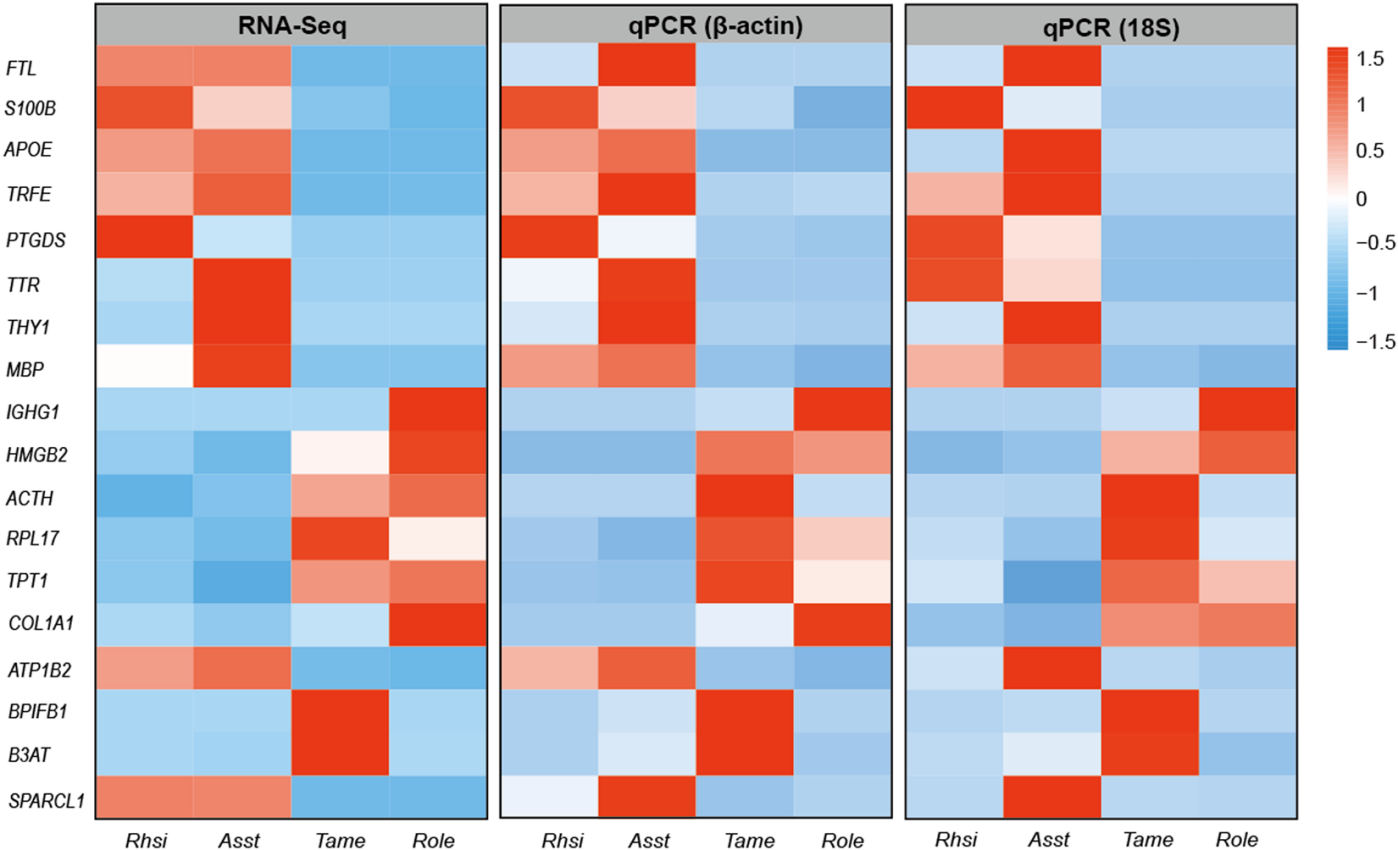
Comparisons of 18 differentially expressed genes by qPCR for technical validation. Heatmap from left to right representing log_2_ fold change expression values from RNA-Seq (rpkm) and qPCR (using β-actin and 18S for normalisation). The correlation coefficient between log_2_ fold change expression values detected by RNA-Seq and qPCR was 0.424 (p < 0.01).

To confirm the reliability of the results obtained by RNA-Seq, we compared the RNA-Seq data of 18 randomly selected genes with the results of qPCR experiments, which were used to determine the changes of mRNA expression of the selected genes among samples from the four bat species. All of the tested target genes showed expression patterns similar to the results obtained by RNA-Seq (Fig. 6). Moreover, significant correlations between log_2_ fold change values detected by RNA-Seq and qPCR indicated that the two independent measurements were consistent (*p* < 0.01). Our qPCR validation confirmed the reliability of the RNA-Seq data.

## Discussion

As was first proposed several decades ago, alterations (or innovations) in gene expression are regarded as an essential means of generating biological diversity^1,2^. Therefore, analyses of DEGs can reveal the molecular mechanisms underlying phenotypic diversity and provide a deeper understanding of the relationship between gene expression patterns and the resultant phenotypes^30,31^. In this study, we performed transcriptomic analysis of the cochleae of four bat species with three different echolocation types using Illumina sequencing technology. Numerous DEGs were identified in the six pairwise comparisons and a series of analyses of DEGs indicated that the molecular differences in the cochleae for CF vs. Click were greater than for CF vs. FM; the smallest difference was found in FM vs. Click. Subsequently, based on the results from downstream analyses of DEGs, two important standpoints were induced as following.

### Different nervous activities in the cochlea of CF bats

The results obtained in this study indicate the presence of different nervous activities in CF bats compared with those in the other bats, as revealed by the six pairwise comparisons and clustering analysis. On the one hand, for the six pairwise comparisons, both GO and KEGG enrichment analyses indicated significantly different nervous system and ion transport activities in the CF bats’ cochleae (*R. sinicus* and *A. stoliczkanus* here). Even those upregulated genes that were significantly enriched in three hearing-related GO terms (biological process) (see Supplementary Table S3) were all nervous system-related genes. On the other hand, clustering analysis revealed a clear set of genes, super-group 1 with 1,504 DEGs, with higher expression levels in CF bats, which were significantly enriched in various hearing-related processes, suggesting their importance in the cochlea of CF bats. Furthermore, a set of 426 genes that were identified as core genes for CF bats also exhibited findings similar to those above.

The important acoustic parameter of dominant frequency provides a lot of vital information, but the dominant frequency differs largely among bat species^32–34^; this is regarded as one of the possible reasons for the differences of gene expression among the different bat species identified in this study. The perception of echolocation signals begins at the hair cells in the cochlea, continues along the auditory nerve and terminates at the auditory cortex of the brain^35^. Several previous studies documented that bats with distinct types of echolocation can respond well to their own acoustic signals via special cochlear adaptations^13,22,36^. In consideration of the fact that CF bats have a much higher dominant frequency (83.15 and 117.87 kHz for *Rhsi* and *Asst*, respectively) than FM (30.67 kHz of *Tame*) and click bats (37.05 kHz of *Role*), these overexpressed genes could constitute an important molecular basis for responses to high-frequency signals in the cochlea of CF bats.

Besides frequency, an alternative explanation for the differences in gene expression among different bat species might be the differences of cochlear morphology (e.g. cochlear size, basilar membrane (BM) length and number of cochlear turns) in the bats with three different types of echolocation^36,37^. The cochlea of bats functions as an apparatus for receiving and processing sounds in the inner ear; its size has been demonstrated to be correlated with echolocation behaviour and its structure has significantly contributed to the diversification of bat species^38,39^. In addition, the mechanical properties of the BM of bats provide the foundation for ultrasonic hearing^40^. Given the critical role of the BM in the organ of Corti, it is expected that the BM might show adaptations for processing ultrasonic echoes. Previous research^21,36,41^ demonstrated that CF bats usually have longer relative BM lengths and a larger number of cochlear turns than FM bats and click bats, which is probably an adaptation to their fine auditory tuning to their CF calls. Hence, in this study, the upregulated genes detected in CF bats, especially those involved in ear development or related pathways, could be functionally important for CF bats.

The most likely explanation for the presence of different nervous system activities in CF bats compared with those in the other bats is that the specialised neurons and their structures have only emerged in CF bats. The frequencies of the bat’s dominant CF components are overrepresented throughout the ascending auditory pathway, from the cochlea up to the auditory cortex, forming an acoustic fovea^23,42^. Our results revealed a large number of genes overexpressed in the cochleae of CF bats that were significantly associated with the generation of neurons and neurogenesis, as well as nervous system development, among others, indicating the functional importance of these genes. The patterns of overexpression of those genes might be due to the different number of specific neurons in the auditory fovea of CF bats, such as a larger population of neurons with sharp frequency tuning to a ultranarrow frequency range in the cochleae of CF bats^22,43–45^. The ultranarrow frequency tuning of these neurons allows bats to detect Doppler shifts of target echoes, for computing the velocity of insects and detecting the rapid Doppler modulations caused by the fluttering of their wings^22,23^. However, FM and click bats using pure FM signals have no such distinct abundance of neurons with specific frequencies^22,24^. Additionally, specialised neurons with different response properties were also found to be present in the auditory cortex of bats with different types of echolocation, which may help us to understand the differences of neurons and nervous activities in the cochleae of different bat species.

### Genes functionally important for laryngeally echolocating bats

We also found a set of genes, super-group 2 with 1,764 DEGs, showing similar expression patterns, namely, upregulation in CF and FM echolocating bats, but downregulation in click bats. This may suggest their functional importance in the cochleae of laryngeal echolocators.

Both CF and FM bats can produce ultrasonic signals via their larynxes, but click bats can emit echolocation calls through clicking of the tongue. These differences in the mechanisms used to produce ultrasonic signals may lead to different mechanisms of perceiving signals in laryngeally and click echolocating bats. Unique and important nervous system-related hearing processes were detected in the cochleae of laryngeally echolocating bats by GO and KEGG enrichment analyses. In addition, previous studies revealed molecular differences between laryngeal and tongue-click echolocation at the sequence level for several important hearing-related genes^46,47^. For instance, the two hearing-related genes *Prestin* and *KCNQ4* were shown to have undergone convergent or parallel evolution in laryngeally echolocating bats with high-frequency hearing, but not in click bats from the genus *Rousettus*^48–50^.

Moreover, the high rate of use of echolocation signals in laryngeally echolocating bats may also be responsible for this particular gene expression pattern in super-group 2. Laryngeally echolocating bats rely heavily on their acoustic signals for survival, in comparison with click bats^37^. Echolocation as one of the most important sensory systems is widely used by laryngeally echolocating bats for orientation, predation and even communication^13^. However, besides biosonar, tongue-click bats also have advanced vision and use visual information more frequently^15,51^. Therefore, acoustic signals are used more frequently by laryngeally echolocating bats than by tongue-click bats. Thus, the selective pressures related to hearing perception might be more stringent in laryngeal echolocators than in tongue-click bats, which may have led to the different expression of related genes.

## Conclusions

For over half a century, echolocating bats have served as a valuable model in neuroscience to elucidate the mechanisms of auditory processing and adaptive behaviour in biological sonar. At the neurobiological level, the auditory system of echolocating bats has been the focus of intense investigation over the last four decades in a range of bat species. Our study sheds light on the molecular mechanisms involved and provides a number of important genes that underlie the process of auditory perception, especially those related to neural transduction in bats employing different types of echolocation. In particular, a super-group of 1,504 genes was identified as having physiological functions that are crucial for CF bats, among which 426 genes were shown to be the most important. Moreover, another super-group of 1,764 genes with functional importance for laryngeally echolocating bats was also identified. Subsequently, differences in biological processes and physiological activities among the different bat species were detected, with significant differences in the nervous system activities in the cochleae of CF bats in particular being revealed. Although only a few bat species were selected for analysis in this study, this work still provides a comprehensive overview of the genetic basis for different types of echolocation in bats and reveals the huge differences in the biological mechanisms employed by bats with distinct hearing traits.

## Materials and Methods

### Ethics statement

According to the regulations of Wildlife Conservation of the People’s Republic of China (Chairman Decree [2004] No. 24), permits are required only for species included on the list of state-protected and region-protected wildlife. None of the bats used in this study are endangered or region-protected species, so no specific permission is required. All animal experimental procedures were approved by the National Animal Research Authority of Northeast Normal University, China (approval number: Nenu-20080416), and the Forestry Bureau of Jilin Province, China (approval number: [2006]178). All efforts were made to minimise the suffering of the animals.

### Sample collection

All animals used in this study were caught during July 2016 in Yunnan Province, China. To avoid any influence of sex-related differences, only females were selected for inclusion in the study. All individuals were kept under similar environmental conditions, including temperature and humidity, and then sacrificed at the same time of day. Three biological repeats were included for each species and 12 individuals were collected in total. A pair of cochlear tissues from each individual were collected and flash-frozen in liquid nitrogen as soon as possible, followed by placement in a −80 °C freezer until processing for total RNA isolation.

### Echolocation call recording and analysis

Echolocation calls were recorded with a real-time ultrasonic detector (UltraSoundGate 116; Avisoft Bioacoustics, Berlin, Germany) for each individual in the field, with a condenser ultrasound microphone [Ultrasoundgate CM16/CMPA, a flat frequency response between 10 Hz and 200 kHz (±3 dB)] located at a distance of approx. 2 m from the bat. The recordings with a high signal-to-noise ratio (>40 dB) were analysed using the software Avisoft SasLab Pro [length of fast Fourier transformation (FFT): 512 points with 75% overlap, spectral resolution: 488 Hz; Avisoft Bioacoustics]. For each individual under resting conditions, the dominant frequencies of 15 high-quality calls were analysed for their appropriate dominant harmonic from the power spectra of a call, and the mean value was used in the analysis. Then, spectrogram representations (frequency × time) of echolocation calls for the four bat species were obtained to reveal the differences in their call types (Fig. 1a).

### RNA extraction

Cochleae subjected to identical homogenisation were used for each sample library. Total RNA was isolated using TRIzol reagent (Life Technologies Corp., Carlsbad, CA, USA), in accordance with the manufacturer’s protocol. The quantity and quality of total RNA were measured using an Agilent 2100 Bioanalyzer (Agilent Technologies, Palo Alto, CA, USA) and gel electrophoresis. RNA samples of the same volume and concentration were used during the step of converting mRNA into cDNA. Three paired-end cDNA libraries of each bat species were generated using the mRNA-Seq assay. In total, 12 cDNA libraries were prepared at an equimolar ratio for transcriptome sequencing on the Illumina Hiseq 4000 platform. The raw sequence data generated were deposited into the NCBI Sequence Read Archive database (SRA run accession numbers: *R. sinicus*: SRS3009150, *A. stoliczkanus*: SRS3011421, *T. melanopogon*: SRS3011514, *R. leschenaultii*: SRS3011407).

### Transcriptome assembly and functional annotation

The raw reads were filtered using three criteria: removing reads with adaptors; removing reads with unknown “N”; and removing low-quality reads containing more than 50% low-quality bases (Q-value ≤ 20). To construct a common and powerful reference transcriptome for the comparative analyses, all high-quality raw reads from 12 individual cDNA libraries were used for *de novo* assembly by Trinity software^52^ with the default parameters. The assembled contigs with a minimum length of 200 bp were used for further analyses. Then, the CD-Hit program^53^ was used to reduce sequence redundancy of the transcriptome with the default parameter settings. All of the remaining contigs are described as unigenes in the following text.

Basic annotations of unigenes include protein functional annotation, KOG functional annotation, Gene Ontology (GO) and pathway annotations. In detail, we used BLASTX (http://www.ncbi.nlm.nih.gov/BLAST/) with an E-value threshold of 1e−5 on the NCBI nonredundant protein (Nr) database (http://www.ncbi.nlm.nih.gov), the Swiss-Prot protein database (http://www.expasy.ch/sprot), the KOG database (http://www.cubi.nlm.nih.gov/KOG) and the Kyoto Encyclopedia of Genes and Genomes (KEGG) database (http://www.genome.jp/KEGG). The GO annotation of unigenes was conducted using Blast2GO software^54^ and their functional classification was performed using WEGO software^55^.

### Differential expression analysis

In consideration of the differences of library size, we first performed inter-sample normalisation. To compare the gene expression profiles, raw reads from the 12 cDNA libraries were separately mapped back to the reference transcriptome (length > 500) using Bowtie2^56^ implemented in Trinity^52^, with two critical parameters: fewer than five mismatches and no gaps. Unique mapped reads were quantified into counts for each unigene. RPKM values (reads per kilobase of transcript per million mapped reads) were applied to determine the level of expression of each gene^57^.

Then, the correlation coefficient between each pair of replicates for the four bat species was calculated using R package (version 2.16.2) to evaluate the reliability of the experimental results as well as the operational stability. To determine the separation of expression patterns across samples, PCA was performed on the levels of all unigenes using R package (gmodels, version 3.4.1).

As RNA-Seq is less accurate at detecting DEGs with low expression levels, unigenes from each species with RPKM > 1 were retained for the following analyses. DEGs among the four species were evaluated using edgeR^58^, which is particularly powerful for DEGs detection^59^; thus, a total of six pairwise tests were conducted. In consideration of the general divergence among bat species and that the detection of DEGs would be more accurate for those with a greater difference in expression, a threshold of fold change ≥ 4 was applied for all pairwise comparisons in this study. Hence, to create a list of high-confidence DEGs for further analyses, the following stringent criteria were used: fold change ≥ 4, namely |log_2_ fold change| ≥ 2 and adjusted *p* value ≤ 0.001^60^.

Visualisation of the DEGs in the four bat species was achieved by creating heatmaps with the GPLOTS package of R (http://www.r-project.org/).

### GO category and KEGG pathway enrichment analyses

Downstream functional classification was achieved through the integrated localisation of GO^61^ and KEGG pathway databases^62^. All *p* values were computed using the hyper-geometric test, and multiple test correction was performed using the Benjamini–Hochberg method^60^ based on an FDR (false discovery rate) cut-off of 0.001.

### DEGs clustering analysis

Given that the dominant frequencies exploited by echolocating bats are reasonable from an acoustics perspective, clustering analysis was performed based on the gene expression patterns in the four bat species ordering them by their dominant frequency from low to high. The order of species was *Tame*, *Role*, *Rhsi* and *Asst* for clustering analysis of DEGs (*Tame*, *Role*, *Rhsi* and *Asst* represent *T. melanopogon*, *R. leschenaultii*, *R. sinicus* and *A. stoliczkanus*, respectively). First, we constructed a set combining the six groups of DEGs, including 10,630 of them. Subsequently, those DEGs were clustered into several clusters based on their expression level using the SOM clustering algorithm implemented in EXPANDER 7.2 (Expression Analyzer and DisplayER)^63,64^. These clusters were then refined using homogeneity parameters and reclustering^65^. Homogeneity is the degree of similarity of elements in the same cluster; a higher level of homogeneity represents better clustering of genes in a profile^66,67^. To obtain tight groups of highly similarly expressed genes among the four bat species, we used 0.9 as the criterion of homogeneity for the subsequent cluster selection. For DEGs that were grouped in the same cluster, downstream functional classifications were achieved through the integrated localisation of GO^61^ and KEGG pathway databases^62^.

### Genes important for CF bats

Considering the specific echolocating phenotype and relatively high dominant frequency in CF bats, the genes that are consistently upregulated in the two CF bats relative to the levels in click and FM bats could be the most important for CF bats. We thus extracted the genes upregulated in CF bats that overlapped among the four CF-related pairwise comparisons: *Rhsi* vs. *Role, Asst* vs. *Role, Rhsi* vs. *Tame* and *Asst* vs. *Tame*. Then, we performed GO and KEGG pathway enrichment analyses to obtain insights into which pathways related to hearing behaviour are enhanced in the cochleae of CF bats.

### Validation of sequencing data by qPCR

To confirm the expression patterns observed in our RNA-Seq analyses, 18 randomly selected target genes and two housekeeping genes were used. The gene names and the sequences of their primer pairs are listed in Supplementary Table S7. Three replicates were analysed per species using qPCR on the Applied Biosystems StepOne Real-Time PCR System (Applied Biosystems). Complementary DNAs (cDNAs) were synthesised using the same RNA samples as used for RNA-Seq and 1 µg of total RNA was reverse-transcribed using reverse transcriptase (Trans, Beijing, China). The TransStart Top Green qPCR SuperMix (Trans) was used for qPCR reactions. The final reaction volume of 20 μl included 1 μl of cDNA, 10 μl of 2 × TransStart Top Green qPCR Supermix, 0.4 μl of each primer, 0.4 µl of 50× ROX reference dye and 7.8 μl of ddH_2_O. Then, PCR was performed under the following conditions: pre-denaturation at 94 °C for 30 s; then 40 cycles of 94 °C for 15 s, 60 °C for 1 min and 72 °C for 10 s, with data collection after each cycle, followed by the application of a melting curve. The efficiencies of amplification of the 18 target genes and two housekeeping genes were all between 90% and 100%. The relative expression levels of each target gene were calculated against two references genes, β-actin and 18S, by using the 2^−ΔΔCT^ method^68^. Regression analysis was performed to compare the expression values from the qPCR with the RNA-Seq results.

## Electronic supplementary material

Dataset 1

Dataset 2

Dataset 3

Dataset 4

Dataset 5

Dataset 6

Dataset 7

Dataset 8

## Data Availability

All data generated or analysed during this study are included in this paper (and its Supplementary Information files).
