# Supplementary material for: Comparative cochlear transcriptomics of echolocating bats provides new insights into different nervous activities of CF bat species
Source: Sci Rep. 2018 Oct 29;8:15934. doi: 10.1038/s41598-018-34333-7 (PMC6206067; doi:10.1038/s41598-018-34333-7)
Supplement: Supplementary file 8 — Dataset 8 [file 41598_2018_34333_MOESM8_ESM.pdf]

Comparative cochlear transcriptomics of echolocating bats provides new insights into different nervous activities of CF bat species

Hui Wang, Hanbo Zhao, Xiaobin Huang, Keping Sun & Jiang Feng

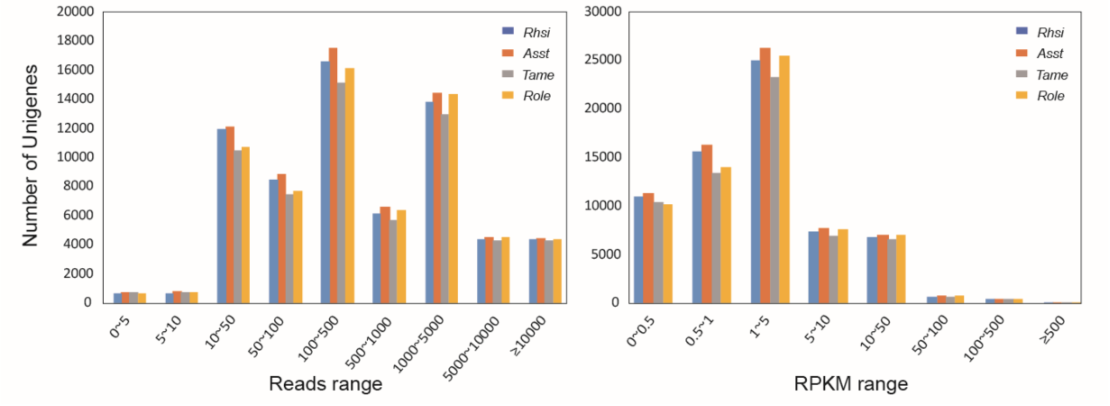

Figure S1. Interval distribution of unigene expression abundance vs. read range (left) and RPKM range (right).

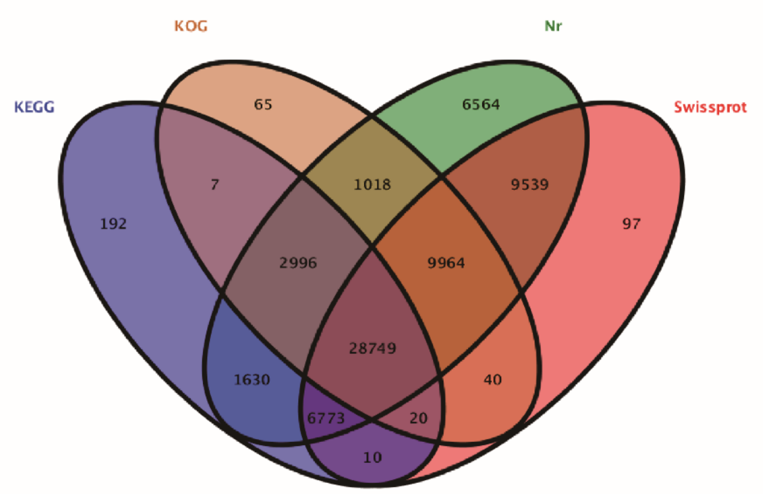

Figure S2. Venn diagram of Nr, Swiss-Prot, KOG and KEGG databases, showing homology sequence numbers of the unigenes in the four different databases.

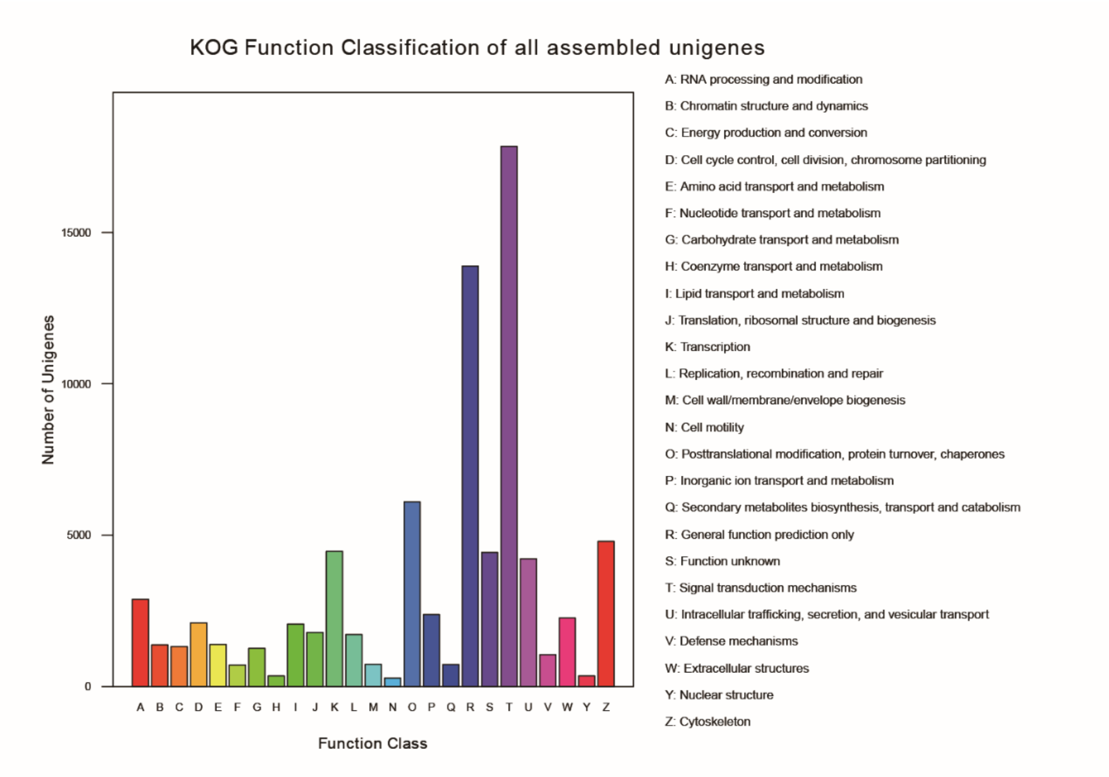

Figure S3. KOG functional classification of the unigenes. A total of 25 categories were obtained.

The x-axis indicates the categories and the y-axis indicates the number of unigenes.

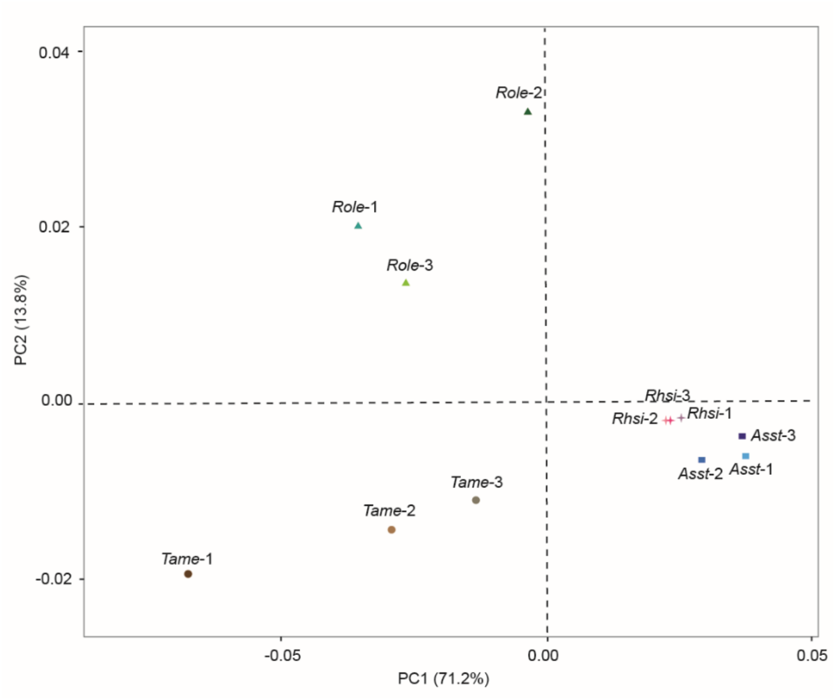

Figure S4. Principal component analysis (PCA) of the transcriptome of four bat species. The numbers in parentheses represent the proportion of variance explained by that principal component. PC1 and PC2 represent the top two dimensions of the genes showing differential expression among these samples, which account for 71.2% and 13.8% of the expressed genes, respectively.

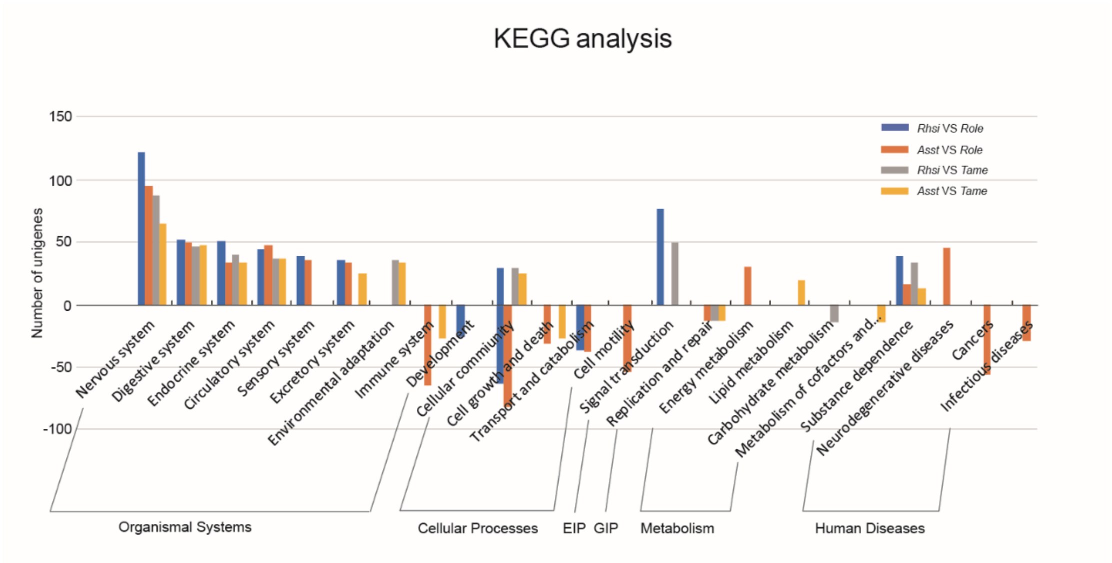

Figure S5. KEGG pathways significantly (p < 0.01) enriched for DEGs from four comparisons: *Rhsi* vs. *Role* (blue), *Asst* vs. *Role* (orange), *Rhsi* vs. *Tame* (grey) and *Asst* vs. *Tame* (yellow). EIP stands for environment information process; GIP stands for genetic information process.

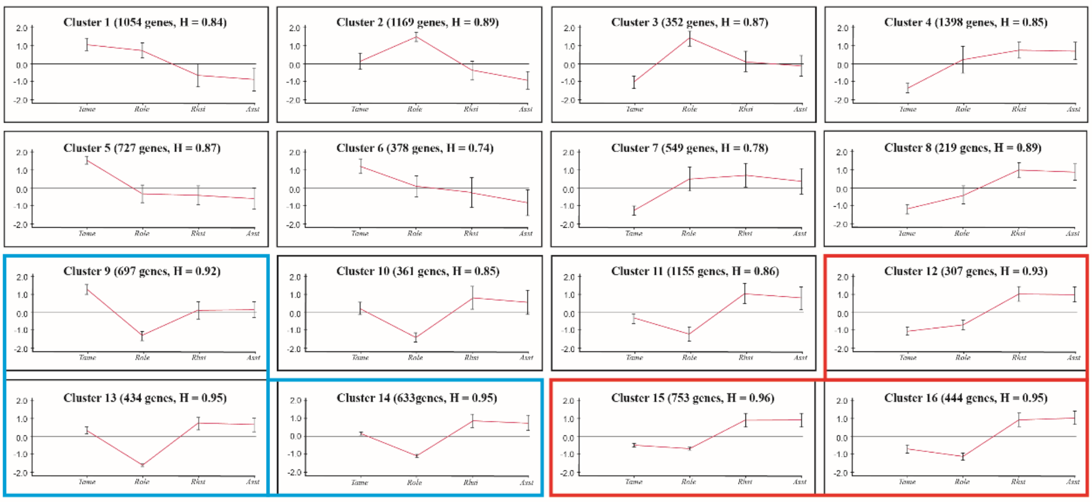

Figure S6. Sixteen clusters of 10,630 DEGs based on their gene expression patterns in the order of their dominant frequency from low to high: *Tame*, *Role*, *Asst* and *Rhsi*. In red super-group 1 (clusters 12, 15 and 16) and blue super-group 2 (clusters 9, 13 and 14), colours represent gene clusters displaying similar expression with homogeneity (H) values larger than 0.9. The number of genes in each cluster and the H value are shown on the top of the curve. Each cluster graph displays the mean pattern of expression (red lines) of the genes in it. The x-axis represents species in order of their dominant frequency from low to high and the y-axis represents log_2 fold change in gene expression.
